# Therapeutic Vitamin D Supplementation Following COVID-19 Diagnosis: Where Do We Stand?—A Systematic Review

**DOI:** 10.3390/jpm12030419

**Published:** 2022-03-08

**Authors:** Angelina Bania, Konstantinos Pitsikakis, Georgios Mavrovounis, Maria Mermiri, Eleftherios T. Beltsios, Antonis Adamou, Vasiliki Konstantaki, Demosthenes Makris, Vasiliki Tsolaki, Konstantinos Gourgoulianis, Ioannis Pantazopoulos

**Affiliations:** 1 Faculty of Medicine, School of Health Sciences, University of Patras, 26504 Rion, Greece; bania.ang@gmail.com; 2 School of Medicine, University of Crete, 71003 Heraklion, Greece; kpitsikakis.00@gmail.com; 3 Department of Emergency Medicine, Faculty of Medicine, School of Health Sciences, University of Thessaly, 41110 Larissa, Greece; beltsioseleftherios@gmail.com (E.T.B.); pantazopoulosioannis@yahoo.com (I.P.); 4 Department of Anesthesiology, Faculty of Medicine, School of Health Sciences, University of Thessaly, 41110 Larissa, Greece; mmermiri@gmail.com; 5 Department of Radiology, Faculty of Medicine, School of Health Sciences, University of Thessaly, 41110 Larissa, Greece; antonadamou@gmail.com; 6 Department of Anesthesiology, General Hospital of Limassol, Limassol 4131, Cyprus; vasokonstant@yahoo.gr; 7 Critical Care Department, Faculty of Medicine, School of Health Sciences, University of Thessaly, 41110 Larissa, Greece; demomakris@uth.gr (D.M.); vasotsolaki@yahoo.com (V.T.); 8 Department of Respiratory Medicine, Faculty of Medicine, School of Health Sciences, University of Thessaly, 41110 Larissa, Greece; kgourg@uth.gr

**Keywords:** COVID-19, vitamin D, hospitalization, ICU admission, intubation, mortality

## Abstract

Vitamin D has known immunomodulatory activity and multiple indications exist supporting its potential use against SARS-CoV-2 infection in the setting of the current pandemic. The purpose of this systematic review is to examine the efficacy of vitamin D administered to adult patients following COVID-19 diagnosis in terms of length of hospital stay, intubation, ICU admission and mortality rates. Therefore, PubMed and Scopus databases were searched for original articles referring to the aforementioned parameters. Of the 1376 identified studies, eleven were finally included. Vitamin D supplements, and especially calcifediol, were shown to be useful in significantly reducing ICU admissions and/or mortality in four of the studies, but not in diminishing the duration of hospitalization of COVID-19 patients. Due to the large variation in vitamin D supplementation schemes no absolute conclusions can be drawn until larger randomized controlled trials are completed. However, calcifediol administered to COVID-19 patients upon diagnosis represents by far the most promising agent and should be the focus of upcoming research efforts.

## 1. Introduction

The ongoing COVID-19 pandemic proven a major challenge both for the scientific community and society in general, resulting in millions of deaths worldwide [1]. Despite the immunization of a large percentage of the world population [1], predominantly in first-world countries, SARS-CoV-2 and its variants remain a significant cause of morbidity and mortality. In the absence of SARS-CoV-2-specific pharmacological agents, drug repurposing has emerged as the only available treatment strategy. Remdesivir plus dexamethasone, immunomodulatory agents and, more recently, monoclonal antibodies are approved under Emergency Use Authorization for various severity stages of COVID-19 [2], but efforts for more, largely available and safe drugs are continuous.

Vitamin D is a fat-soluble vitamin, regulating circulating calcium and phosphate levels with an important role in bone homeostasis. The active form of vitamin D is 1,25(OH)_2_D3 (calcitriol) and its biosynthesis includes the conversion of skin 7-dehydrocholesterol to pre-vitamin D3 and then vitamin D3 (cholecalciferol) in the presence of ultraviolet sun radiation [3,4], followed by two steps of hydroxylation to 25(OH)D3 (calcifediol) by the liver and finally to 1,25(OH)2D3 by the kidney. The vitamin D receptor (VDR) acts as a transcription factor and alongside the retinoid X receptor (RXR) binds on a DNA motif on a variety of human tissues [5], regulating the epigenome and expression of thousands of genes and gene networks [6], involved in mineral, bile acid and exogenous compound metabolism, cell differentiation and immune response [7].

Vitamin D deficiency, defined by the Endocrine Society [8] as 25(OH)D3 below 20 ng/mL and vitamin D insufficiency, defined as 25(OH)D3 of 21–29 ng/mL are highly prevalent findings among the general population, linked to rickets in children and osteomalacia and osteoporosis in adults, as well as diabetes, cardiovascular disease, auto-immune disorders, cancer, hepatitis B and C, allergies, asthma and respiratory tract infections [4,9].

In the current setting, vitamin D has been shown to exert immunomodulatory actions in SARS-CoV-2 infection [10,11]. More specifically, it increases the expression of defensins and cathelicidin (LL-37), an endogenous antimicrobial [12], as well as other antiviral agents involved in the TNF-a [13], IFN-γ [14] and NF-κB [15] pathways. It also reduces inflammation, and thus the risk to develop the potentially fatal Cytokine Storm Syndrome, by inhibiting the Th1 response and the production of inflammatory cytokines [14], while enhancing the production of anti-inflammatory cytokines [14]. Its role as a potential immunomodulatory agent is further supported by its capacity to increase regulatory T lymphocytes [16], which are significantly decreased in the setting of COVID-19 [17].

Vitamin D has been hypothesized to intervene in the mechanism by which COVID-19 infection induces a hypercoagulative state [18,19], thus increasing the risk for thrombosis, as well as results to the Acute Respiratory Distress Syndrome (ARDS). It is known that SARS-CoV-2 utilizes the angiotensin converting enzyme 2 (ACE2) receptor [20], thus downregulating it. This results in excessive accumulation of angiotensin II, the substrate of ACE2, which can lead to ARDS [21]. Serum vitamin D levels have been found to be inversely correlated with the Renin-Angiotensin-Aldosterone System activation [22,23], meaning that in COVID-19 patients with vitamin D deficiency, the increase of angiotensin may facilitate progress to ARDS. Conversely, vitamin D can protect from ARDS by lowering renin and increasing ACE2 expression [24].

Based on these data and thanks to their safety profile, availability and low cost, vitamin D supplements are currently used as an off-label pharmacological agent for the treatment of SARS-CoV-2 infection, while their efficacy has been examined in multiple studies with varying results. In this systematic review, we aim to summarize the most recent evidence regarding the therapeutic role of vitamin D on severe COVID-19 outcomes (length of hospital stay, mechanical ventilation, mortality) in adult populations.

## 2. Materials and Methods

### 2.1. Protocol

The protocol for this systematic review is registered in the International Prospective Registry of Systematic Reviews, PROSPERO, under the ID: PROSPERO2021 CRD42021281646 and is fully available online at https://www.crd.york.ac.uk/prospero/display_record.php?ID=CRD42021281646 (Last accessed: 18 February 2022; 19:01:38 EET).

### 2.2. Literature Search

Two investigators (A.B. and K.P.) individually performed an electronic search of the PubMed (MEDLINE) and Scopus databases to identify relevant studies, based on the predetermined inclusion and exclusion criteria. Disagreements between the two authors were resolved by discussion between them or with the help of a third investigator (G.M.), when necessary. The search algorithms, fully available in the Supplementary File Document S1, consisted of the terms ‘vitamin D’ and ‘COVID-19’ and their derivatives, as well as the Boolean operators ‘AND’ and ‘OR’. The references of previous systematic reviews and meta-analyses were also screened for additional original studies. Only articles fully available in the English language were included in this review. The last literature search was performed on 28 September 2021.

### 2.3. Inclusion and Exclusion Criteria

Original articles, restricted to randomized controlled trials, prospective and retrospective observational studies, case-control studies and case series with at least ten participants were included in this systematic review. No restriction on publication date was imposed. The studies pertained to the post-diagnosis administration of any form of vitamin D to adult (>18 years of age) patients diagnosed with COVID-19. Our studied outcomes were: duration of hospitalization, need for mechanical ventilation/intubation, ICU admission and all-cause mortality.

Studies on the chronic supplementation with vitamin D and studies focusing on paediatric populations were excluded from this systematic review. Congress abstracts, letters to the editor, case reports, case series of less than ten patients, ecological studies, reviews and meta-analyses were also excluded.

Jevalikar et al. [25] included a small number of children in their cohort. Here we only report findings based on the data relevant to vitamin D administration in a sub-population of the initial cohort, but the presence of children in this sub-group is not specified.

### 2.4. Data Extraction

Using a pre-determined data table, two of the authors (A.B. and K.P.) performed the data extraction. The following data were extracted: First Author’s Name, Month and Year of Publication, Study Design, Vitamin D Administration Scheme, Control Method, Population Size and Number of Participants in each group, Male to Female Ratio, Mean Age, Presence Of Comorbidities (Hypertension, Cancer, Myocardial Infarction, Diabetes Mellitus, Chronic Obstructive Pulmonary Disease, Chronic Kidney Disease, Obesity), Baseline And Post-Intervention Serum Vitamin D Levels in each group, Mortality, Length Of Hospital Stay, ICU Admissions and Intubation events in each group, Mortality Time Point and Length of Follow-Up. 

### 2.5. Quality Assesment

Quality scoring was performed using the Cochrane Risk of Bias (RoB) [26] tool for the Randomized Controlled Trials and the Methodological Index For Non-Randomized Studies (MINORS) [27] for the observational studies. The RoB tool calculates the risk of bias accounting for the randomization process, the deviations from the intended interventions, potential missing data, the outcome measuring methods and the selection of the reported result. The MINORS tool evaluates twelve factors, relevant to the aim and design of the study, patient selection and grouping, follow-up, potential size calculation and result assessment and analysis on a scale of 0–24.

## 3. Results

### 3.1. Search Results

**Figure 1:**
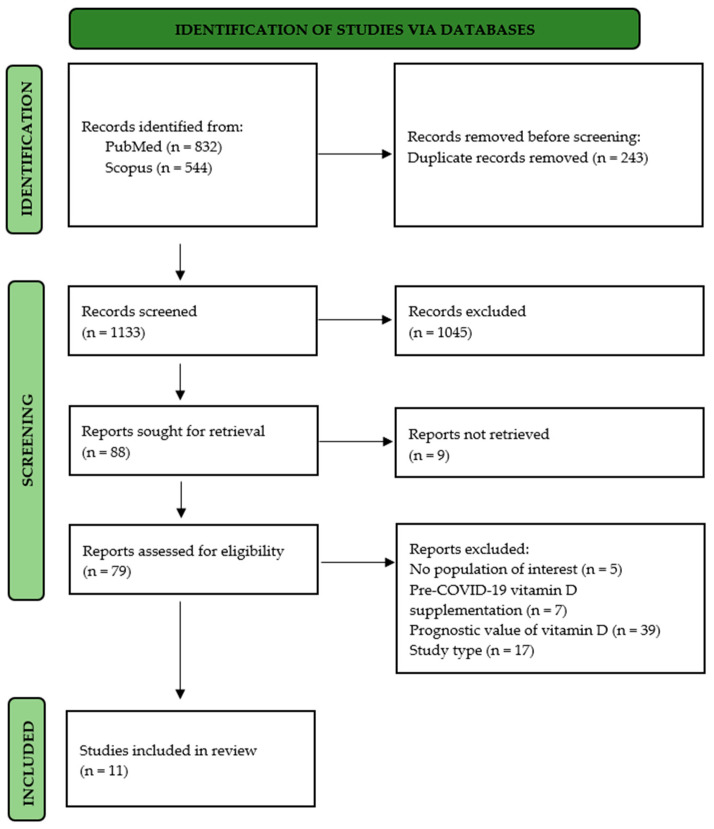
PRISMA flow diagram.

The literature search yielded a total of 1376 articles (832 on PubMed and 544 on Scopus), among which 189 were selected to be evaluated as full texts. Finally, a total of 11 [25,28,29,30,31,32,33,34,35,36,37] articles fully met our inclusion criteria and were included in this systematic review (Figure 1).

### 3.2. Study Characteristics

The studies were published from October 2020 to September 2021 and were conducted in four different continents. Three studies took place in Spain [31,33,34] and one in each of the following countries: France [28], USA [32], Brazil [35] Turkey [30], Singapore [36], Saudi Arabia [29], India [25] and Egypt [37]. The majority [25,28,30,31,33,36,37] were single-center, while in four studies patients from two [35], three [29,32] or five [34] centers were recruited. Our study collection includes four randomized controlled trials [29,32,33,35], one non-randomized controlled trial [28] and six observational cohort studies [25,30,31,34,36,37], among which two [25,31] were reported as prospective and three [34,36,37] as retrospective.

All patients examined in the aforementioned studies were hospitalized for COVID-19 infection. Vitamin D deficiency was not always a prerequisite for inclusion in the studies. A few studies focused on specific subpopulations of COVID-19 patients. More specifically, Güven et al. [30] reported only on vitamin D deficient (25(OH)D3 < 12 ng/mL) patients who had already been admitted to the ICU. In terms of age and comorbidities, Nogues et al. [31] studied high risk patients, i.e., with severe COVID-19 and/or comorbidities, Tan et al. [36] included only patients of age 50 or older, Soliman et al. [37] selected elderly (>60 years of age) vitamin D deficient (<20 ng/mL) Type II diabetics, while Annweiler et al. [28] focused on frail elderly inpatients at a geriatric acute care unit.

The studies and their characteristics are presented in Table 1.

### 3.3. Interventions

The administered substance, its administration route and dosing scheme varied significantly among studies. Seven of them investigated the effect of vitamin D_3_ (cholecalciferol), administered daily per os [29,36], as a high single oral dose of 60,000 IU [25], 80,000 IU [28] or 200,000 IU [35] or as an intramuscular injection of 200,000 [37] or 300,000 IU [30]. Three studies [31,33,34] applied various regimens of oral 25(OH)D3 (calcifediol) and one trial [32] used oral 1,25(OH)_2_D_3_ (calcitriol) at 0.5μg/day for 14 days.

With two exceptions [29,36], the intervention and control groups did not receive any further treatments other than the appropriate standard of care of their centers or placebo. However, in a retrospective study by Tan et al. [36], the intervention group received 1,000 IU/day oral vitamin D3, 150 mg/day oral magnesium and 500 mcg/day oral vitamin B12 for a median interval of 5 days. Finally, in a randomized controlled trial by Sabico et al. [29] both groups received oral vitamin D3, but at different doses (5000 IU vs. 1000 IU).

No severe adverse effects related to this treatment were observed in any of the studies.

### 3.4. Length of Hospital Stay

Out of 10 studies of vitamin D-supplemented vs. vitamin D-non-supplemented patients, three reported on the length of hospitalization. Neither a single dose of 300,000 IU of intramuscular vitamin D_3_ [30], a single dose of 200,000 IU of oral D3 [35] or a 14-day regimen of 0.5 μg 1,25(OH)2D3 per day [32] managed to affect the duration of hospital stay for the intervention group [9(6–16) vs. 9(5–17), *p*-value = 0.649, 7.0(4.0–10.0) vs. 7.0(5.0–13.0) days, *p*-value = 0.59, 5.5 ± 3.9 vs. 9.24 ± 9.4 days, *p*-value = 0.14 respectively]. Additionally, no difference in hospitalization duration was observed between the 5000 IU and the 1000 IU oral D3 group in the randomized controlled trial by Sabico et al. [29] [6(5–8) vs. 7(0–10), *p*-value = 0.14] (Table 2).

### 3.5. Need for Intubation and ICU Admission

Ten out of eleven studies provided data regarding either the need for intubation and mechanical ventilation (three studies) or intensive care admission (four studies) or both (two studies). Entrenas-Castillo et al. [33], explored the effect of a regimen comprised of 0.532 mg oral 25(OH)D3 on the day of admission followed by 0.266 mg on the 3rd and 7th day and then weekly until discharge or ICU admission in a randomized controlled trial. Of 50 patients in the intervention arm, only one required ICU admission, in contrast to the 13/26 patients from the control group (*p*-value < 0.001).

A similar dosing scheme (0.532 mg oral 25(OH)D3 on day 1 plus 0.266 mg on days 3, 7, 15, and 30) was later investigated by Nogues et al. [31], in a large prospective study of 838 high-risk COVID-19 patients. ICU admission was necessary for 21% of the patients in the control group, compared to 4.5% in the intervention group (OR = 0.18 (0.11–0.29), *p*-value < 0.001), showing an 87% risk reduction following adjustment for age, gender, baseline vitamin D levels and comorbidities [OR = 0.13, (0.07–0.23), *p*-value < 0.001]. A statistically significant difference in vitamin D levels between ICU and non-ICU patients was also noted [10 (7–14) ng/mL vs. 13 (8–23) ng/mL, *p*-value < 0.001].

Tan et al. [36], evaluated the combination of vitamin D, vitamin B12 and magnesium in a retrospective study of 43 patients over 50 years of age. The combination therapy was shown to significantly (*p*-value = 0.006) reduce the need for any form of oxygenation therapy. Specifically, 3/17 treated patients required oxygen therapy (including 1 in the ICU), compared to 16/26 non-treated ones (including 8 in the ICU). A subgroup analysis focusing on 30 non-diabetic patients aged 50–60 years was later performed and failed to show a statistically significant difference in oxygenation needs [25% vs. 58.3%, *p*-value = 0.197 and 12.5% vs. 41.7% in regard to ICU admission].

The remaining eight studies, among which the trial of 5000 IU vs. 1000 IU of oral vitamin D3 by Sabico et al. [29] did not show a statistically significant difference in ICU admission [25,29,32,35] and need for intubation [30,32,34,35,37] between study groups (Table 2).

### 3.6. Mortality

All eleven studies reported on the in-hospital mortality of COVID-19 patients. In a multi-center retrospective analysis of 537 patients by Alcala-Diaz et al. [34], 79 patients had received 0.532 mg of oral 25(OH)D3 on day 1 followed by 0.266 mg on day 3 and 7 and then weekly until hospital discharge or ICU admission. Mortality rates among these patients were significantly lower than those of the control group [5% versus. 20%, *p*-value < 0.001, OR = 0.22 (0.08–0.61), *p*-value < 0.01]. Given that all intervention group patients were from the same center and the patients in the control group had a greater comorbidity burden and worse clinical image upon admission, an analysis adjusted for age, center, CURB-65, ARDS at admission, neutrophil/lymphocytes ratio and comorbidities followed and still demonstrated the favorable position of the intervention group in terms of mortality [OR = 0.16 (95%CI = 0.03–0.80), *p*-value = 0.02]. The elderly (>65 years) subgroup with oxygen saturation <96% also greatly benefited from calcifediol administration [OR 0.06 (0.04–0.8), *p*-value = 0.04].

Nogues et al. [31] also attributed a reduction of death rates to 25(OH)D3 administration both in the initial [4.7% vs. 15.9%, OR: 0.26 (0.15–0.43), *p*-value < 0.001] and the adjusted analysis for age, gender, vitamin D levels and comorbidities [OR = 0.21; (95%CI, 0.10–0.43)], which translates into a 70% mortality risk reduction. Baseline vitamin D levels were greater in survivors compared to non-survivors [13 (8–22.7) ng/mL vs. 9 (6–13.5) ng/mL, *p*-value < 0.001].

In this study, 53 of 82 patients from the control group who required intensive care were started on the 25(OH)D3 regimen upon ICU admission. A sub-analysis of a total of 102 ICU COVID-19 patients was then performed. Interestingly in these patients, administration of vitamin D upon initial hospital admission was associated with lower mortality than initiation of supplementation upon ICU admission, while never receiving vitamin D at any point of the disease course had the worst prognosis. However, these differences in mortality were considered statistically insignificant (10.0% vs. 28.3% vs. 31% respectively).

No other study observed significantly different death rates among study groups (Table 2). 

### 3.7. Quality Assessment and Risk of Bias

**Figure 2:**
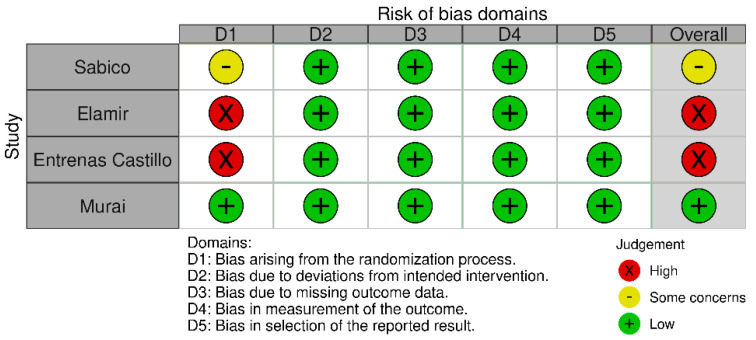
Risk of Bias of Randomized Controlled Trials.

The bias risk for the randomized controlled trials varied significantly, as seen in Figure 2. One study [35] is marked as low-risk, one [29] as moderate risk and two [32,33] as high risk, with concerns arising mainly form the randomization process bias.

The quality of the comparative observational studies ranged from 17 to 22 out of 24, based on the MINORS tool, which translates into moderate and high quality in five [28,30,34,36,37] and two [25,31] studies respectively. MINORS scores for each individual study are reported in Table 3.

## 4. Discussion

To our knowledge, this is the largest and most updated systematic review focusing exclusively on post-COVID-19 diagnosis administration of vitamin D, having included more recent articles compared to previous work. Thus, we have distinguished the therapeutic administration of vitamin D in hospitalized patients following COVID-19 diagnosis from chronic vitamin D supplementation for unrelated purposes.

The aim of this systematic review was to explore the impact of vitamin D administration on important parameters of COVID-19 disease course, such as length of hospital stay, ICU admissions and mortality. Of the four studies mentioning vitamin D and hospitalization duration, none managed to prove an association. Moreover, the majority of studies did not observe significant differences in the need for intubation, ICU admission or mortality, since only four out of eleven studies finally support vitamin D administration to prevent one or multiple among these unfavorable outcomes.

Evidence in favor of the use of vitamin D were identified in one randomized controlled trial of 76 patients [33], one large multi-center prospective study of 838 participants [31] and two retrospective studies [34,36] of 537 and 43 patients respectively. The observational studies, which lacked randomization, performed linear regression analyses adjusted for the confounders that were of statistical significance between the two groups and their results remained consistent with the initial findings.

It is possible that the active substance used in each study could have determined its results. Administration of vitamin D3 or 1,25(OH)_2_D3 alone in any form or dose failed to improve any of the outcomes. On the contrary, three out of eleven studies used 25(OH)D3 and all of them reached statistical significance regarding ICU admission, mortality or both. They all took place in Spain and employed a very similar intervention scheme, comprised of an initial oral dose of 0.532 mg 25(OH)D3 followed by 0.266 mg on days 3, 7, 15 and then weekly [33,34] or on days 3, 7, 15 and 30 in the case of Nogues et al. [31]. The fourth study [36] supporting the use of vitamin D supplements to reduce oxygenation and ICU admission used a triple combination of 1000 IU/day oral vitamin D3, 150 mg/day oral magnesium and 500 mcg/day oral vitamin B12 for a median duration of 5 days.

The active vitamin D substance chosen for administration might be of special importance in the setting of renal or liver disease. As expected from the fact that the activation of vitamin D takes place in these tissues, there is a high prevalence for vitamin D deficiency among patients with renal and liver disease [38,39]. Future studies should thus consider administering the fully activated 1,25(OH)_2_D3 to these subgroups or even 25(OH)D3 in the case of liver failure, to bypass the possibly inadequate intrinsic hydroxylation stages.

A general micronutrient sufficiency was shown to reduce SARS-CoV-2 infection and severe illness in a large meta-analysis [40]. Although most heated discussions revolve around vitamin D, other dietary supplements have also been administrated by clinicians in an off-label basis, thanks to their broad role in immune system function and minimal adverse effect burden. Vitamin C [41] and zinc [42] offered no benefit regarding disease outcomes. Vitamin B12, which was co-administered with vitamin D and magnesium in one of our included studies, might facilitate symptom alleviation in COVID-19 [43]. Curcumin, on the other hand, seems to be a more promising agent, associated with faster recovery and lower mortality in a systematic review of six trials [44].

Since the beginning of the COVID-19 pandemic, the use of vitamin D as a prognostic marker and a therapeutic agent has been debatable. This hypothesis was based on pre-existing knowledge from studies on its association with other respiratory tract infections, summarized in recent systematic reviews and meta-analyses, where vitamin D deficiency was found to increase susceptibility to infection [45], while vitamin D seemed to prevent [46,47] or improve [46] the disease course. Similar to our systematic review, a major source of concern on the reliability of these conclusions is the highly variable form of vitamin D analog, its dose and route of administration employed in each of the analyzed studies.

As far as COVID-19 is concerned, vitamin D status is regularly proven to attain a prognostic value in large recent meta-analyses. Lower vitamin D levels are measured in COVID-19 patients than in healthy individuals [42,48], indicating a possible link with susceptibility to infection. Indeed, vitamin D deficiency increased the odds of contracting SARS-CoV-2 by 80% [49]. When it comes to outcomes, a lower vitamin D status was observed in severe disease cases [48], while deficient COVID-19 patients were at an increased risk for prolonged hospitalization [50], ICU admission [51] and death [50,51], although its effect on mortality is quite debatable [48,52].

In the studies presented in this systematic review, no association was observed between baseline levels of vitamin D and the benefits of vitamin D administration. More specifically, two studies [30,37] recruited vitamin D deficient patients only, but no differences were observed between the intervention and control groups. Among the studies demonstrating significant improvements following vitamin D administration, Nogues et al. [31] was the only one providing data on baseline vitamin D levels and these were similar between groups. In any case, vitamin D status should be taken into consideration in the design of future trials.

When it comes to vitamin D as a treatment option, the effect on outcomes other than length of hospital stay, intubation and mortality have also been investigated. High (60,000–80,000 IU) total doses of vitamin D3 failed to reduce the incidence of severe COVID-19, defined as Ordinal Scale for Clinical Improvement (OSCI) score equal to or greater than 5 both in frail elderly [28] and vitamin-D-deficient patients [25]. Furthermore, the effect of vitamin D on inflammatory markers varied across studies. In the aforementioned prospective study by Jevalikar et al. [25], no difference was observed in the fluctuation of any of the inflammatory markers (D-dimers, CRP, LDH, IL6, Ferritin) between the intervention and control groups. The same was reported by Sánchez-Zuno et al. [53], regarding vitamin D3 treated outpatients in regards to transferrin, ferritin and D-dimers. Among a small group of high-dose D3 supplemented and non-supplemented asymptomatic or mildly symptomatic patients the only significantly different decrease was observed in the fibrinogen levels [54]. On the contrary, a similar trial with mildly-moderately affected patients with vitamin D insufficiency reached statistical significance in all (N/L ratio, CRP, LDH, IL6, Ferritin) measured markers [55]. Vitamin D also facilitated symptom alleviation [53] and viral clearance [54] in one of two studies that reported these outcomes.

The role of vitamin D in the treatment plan against COVID-19 had been discussed in previous systematic reviews and meta-analyses. Our conclusions differ from a meta-analysis published by Pal et al. [56], who considered vitamin D supplementation to be beneficial with regards to COVID-19-related ICU admissions and mortality. This is the largest meta-analysis so far, including 13 studies, five of which are common with the ones presented in this systematic review. The difference in our conclusions may be attributed to the study selection. Pal et al. were able to include additional studies compared to our systematic review after contacting their respective authors for data which were not available in the original studies. However, studies associating COVID-19 outcomes with regular vitamin D supplementation, which were excluded in our methodology, were taken into consideration by Pal et al., who subsequently concluded that it is inferior to vitamin D administration after COVID-19 diagnosis. Finally, that meta-analysis does not take into consideration six of the eleven studies presented here, including the five most recent ones.

Previous systematic reviews and meta-analyses containing smaller subsets of articles have reached varying conclusions. Da Rocha et al. [57] were the first to publish a systematic review including three randomized controlled trials on November 2020. These three trials were also the basis for another systematic review by Stroehlein et al. [58] and a meta-analysis by Bassatne et al. [59]. The general conclusion was that vitamin D may have a therapeutic potential, but due to the insufficient, then available, evidence, the need for more, higher quality trials was highlighted.

Other systematic reviews have used subsets of the aforementioned studies and have reached conflicting conclusions supporting or disregarding the therapeutic value of vitamin D in COVID-19. An early meta-analysis by Shah et al. [60] observed the potential of vitamin D to reduce ICU admissions only. A meta-analysis of five studies [41] reported no statistically significant improvements in acute inflammatory markers, ventilation/ICU needs and mortality among patients receiving a variety of different supplementation regimens. This totally contradicts the conclusions of Dramé et al. [61] and Petrelli et al. [62], who express themselves in favor of vitamin D administration to improve all major outcomes. The co-presence of both regular supplementation regimens and post-diagnosis administration as interventions in the included studies is common among many of the systematic reviews and meta-analyses. The common denominator among all these, some of which date back to the very beginning of the pandemic, is the call for large randomized controlled trials. Indeed, the inconsistencies in population selection and more importantly in vitamin D form, dosage and route of administration among the existing studies prevents the extraction of definite conclusions, even two years into the pandemic. Therefore, as we highlight again the necessity for further research, we distinguish calcifediol from all other agents, identifying it as the most promising to be evaluated in upcoming trials.

## 5. Limitations

It has to be noted that, with one exception [35], the clinical trials presented in this review recruited a relatively small number of participants (<100, usually around 50) and this might be a reason for their failure to reach statistical significance.

This systematic review focuses only on the administration of vitamin D following COVID-19 diagnosis to improve important outcomes, such as length of hospital stay, intubation and ICU admission and mortality. Studies discussing the effect of vitamin D as a pre-existing regular supplementation were excluded.

It was also noticed that the included studies employed a highly variable intervention scheme, which consisted of different forms, doses and administration routes of vitamin D which, on one occasion, was co-administered with other agents. It was therefore hypothesized that it could affect the study results making data unsuitable to be pooled or processed in a meta-analysis. Indeed, the form of vitamin D analog seemed to affect outcomes, with 25(OH)D3 being associated with lower ICU admission and mortality, as opposed to vitamin D3 and 1,25(OH)_2_D3.

As about 80% of vitamin D reserves are derived from its biosynthesis in the skin, differences in exposure to UV radiation could influence the results of the included studies. Finally, cases of liver and kidney disease in the studied cohorts might underlie the lack of response to non-activated vitamin D compounds. 

## 6. Conclusions

In this systematic review we have summarized existing knowledge regarding the role of vitamin D on important COVID-19 outcomes indicative of disease severity (length of hospital stay, ICU admission, mortality). Despite the conflicting evidence surrounding the effect of vitamin D across the reviewed studies, we observed 25(OH)D3 (calcifediol) to be by far the most successful agent in reducing intensive care needs and mortality. Therefore, given the insufficient level of evidence of these studies, we are looking forward to larger randomized controlled trials to evaluate calcifediol’s role as an adjuvant to the existing treatment regimens. Finally, given that different SARS-CoV-2 variants are currently spreading worldwide, it could be interesting and useful for further studies to include data on the effect of vitamin D on different variants as well as the patients’ viral load.

## Figures and Tables

**Table 1:** Study Characteristics.

Author, Date of Publication	Study Design	Treatment		Population, Male/Female Ratio, Mean Age, Baseline Vitamin D Levels (ng/mL)	
		Intervention	Control	Intervention	Control
Annweiler [28] Nov-2020	non-randomized clinical trial	80,000 IU oral vitamin D_3_ plus standard care	standard care	16 11/5 85 (IQR = 84–89) NA	32 19/13 88 (IQR = 84–92) NA
Sabico [29] Jun-2021	randomized controlled trial	5000 IU oral vitamin D_3_	1,000 IU oral D_3_	36 21/15 46.3 ± 15.2 21.4 ± 1.2 *	33 13/20 53.5 ± 12.3 25.2 ± 1 *
Güven [30] Sep-2021	observational	300,000 IU of vitamin D_3_ IM	NA	113 69/44 74 (IQR = 60–81) 6.65 (5.06–9.1)	62 36/26 74 (IQR = 60–81) 7.14 (5.17–8.21)
Nogues [31] Sep-2021	prospective	oral 25(OH)D_3_ (532 μg on day one plus 266μg on day 3, 7, 15, and 30) plus standard care	standard care	447 264/183 61.81 ± 15.5 13 (IQR = 8–24)	391 231/160 62.41 ± 17.2 12 (IQR = 8–19)
Elamir [32] Sep-2021	randomized controlled trial	0.5 μg 1,25(OH)_2_D_3_ daily for 14 days oral plus standard care	standard care	25 12/13 69 ± 18 NA	25 13/12 64 ± 16 NA
Entrenas-Castillo [33] Oct-2020	randomized controlled trial	oral 25(OH)D_3_ (0.532 mg), oral calcifediol (0.266 mg) on day 3 and 7, and then weekly plus standard care	standard care	50 27/23 53.14 ± 10.77 NA	26 18/8 52.77 ± 9.35 NA
Alcala-Diaz [34] May-2021	retrospective	oral 25(OH)D_3_ (0.532 mg), then 0.266 mg on day 3 and 7, and then weekly until discharge or ICU admission plus standard care	standard care	79 42/37 69 ± 15 NA	458 275/183 67 ± 16 NA
Murai [35] Mar-2021	randomized controlled trial	single dose of 200,000 IU of oral vitamin D_3_	placebo	119 70/49 56.5 ± 13.8 21.2 ± 10.1	118 63/55 56.0 ± 15.0 20.6 ± 8.1
Tan [36] Nov/Dec 2020	retrospective	1000 IU/d oral vitamin D_3_ and 150 mg/d oral magnesium, and 500 mcg/d oral vitamin B_12_	NA	17 11/6 58.4 ± 7 NA	26 15/11 64.1 ± 7.9 NA
Soliman [37] Sep-2021	prospective	vitamin D_3_ as a single IM (200,000 IU) injection	placebo	40 NA 71.30 ± 4.16 10.4 ± 1.3	16 NA 70.19 ± 4.57 21.17 ± 3.96
Jevalikar [25] Mar-2021	prospective	median total dose of 60,000 IU oral vitamin D_3_	NA	128 NA 45.5 ± 18.2 NA	40 NA 48.8 ± 14.7 NA

IU: International Units, IM: intramuscular, d: day, mg: milligrams, μg: micrograms, ng/mL: nanograms per milliliter, IQR: Interquartile Range, NA: not available. * Originally given at nmol/L, but converted here to ng/mL for consistency.

**Table 2:** Patient Outcomes.

Author	Length of Hospital Stay (Days), Mean ± SD or Median (IQR)		ICU Admission (n/N,%)		Mechanical Ventilation (n/N,%)		All-Cause Mortality (n/N,%)	
	Intervention	Control	Intervention	Control	Intervention	Control	Intervention	Control
Annweiler [28]	NA	NA	all (the study recruited patients already admitted in the ICU)		NA	NA	3/16, 19%	10/32, 31%
Sabico [29]	6 (5–8)	7 (0–10)	2/36, 5.6%	3/33, 9.1%	NA	NA	1/36, 2.8%	0/33, 0%
Güven [30]	9 (6–16)	9 (5–17)	all (the study recruited patients already admitted in the ICU)		44/113, 39%	13/62, 21%	43/113, 38%	30/62, 48%
Nogues [31]	NA	NA	20/447, 4.5%	82/39, 21%	NA	NA	21/447, 4.7%	62/391, 16%
Elamir [32]	5.5 ± 3.9	9.24 ± 9.4	5/25, 20%	8/25, 32%	0/25, 0%	2/25, 8%	0/25, 0%	3/25, 12%
Entrenas-Castillo [33]	NA	NA	1/50, 2%	13/26, 50%	NA	NA	0/50, 0%	2/26, 7.7%
Alcala-Diaz [34]	NA	NA	NA	NA	3/79, 3.8%	26/458, 5.7%	4/79, 5.1%	90/458, 20%
Murai [35]	7.0 (4.0–10.0)	7.0 (5.0–13.0)	16.0 % (9.9–22.5)	21.2% (14.2–29.7)	7.6% (3.5–13.9)	14.4% (8.6–22.1)	7.6% (3.5–13.9)	5.1% (1.9–10.7)
Tan [36]	NA	NA	1/17, 5.9%	8/26, 31%	NA	NA	0/17, 0%	0/26, 0%
Soliman [37]	NA	NA	NA	NA	14/40, 35%	7/16, 44%	7/40, 18%	3/16, 19%
Jevalikar [25]	NA	NA	16/128, 13%	13/40, 33%	NA	NA	1/128, 0.8%	3/40, 7.5%

ICU: Intensive Care Unit, IQR: Interquartile Range, NA: not available.

**Table 3:** MINORS Score for non-randomized trials.

Author	MINORS Score (Out of 24)
Annweiler	18
Guven	18
Nogues	19
Alcala Diaz	17
Tan	18
Jevalikar	22
Soliman	17

## Data Availability

Data are available upon request.

## Supplementary Materials

The following supporting information can be downloaded at: https://www.mdpi.com/article/10.3390/jpm12030419/s1, Document S1: Literature search algorithms.
