# Supplementary material for: Therapeutic Vitamin D Supplementation Following COVID-19 Diagnosis: Where Do We Stand?—A Systematic Review
Source: J Pers Med. 2022 Mar 8;12(3):419. doi: 10.3390/jpm12030419 (PMC8950116; doi:10.3390/jpm12030419)
Supplement: Supplementary file 1 [file jpm-12-00419-s001.zip › jpm-1624726-supplementary.pdf]

PubMed

("vitamin d"[MeSH Terms] OR "vitamin d"[All Fields] OR "ergocalciferols"[MeSH Terms] OR "ergocalciferols"[All Fields]) AND ("covid 19"[All Fields] OR "covid 19"[MeSH Terms] OR "covid 19 vaccines"[All Fields] OR "covid 19 vaccines"[MeSH Terms] OR "covid 19 serotherapy"[All Fields] OR "covid 19 serotherapy"[Supplementary Concept] OR "covid 19 nucleic acid testing"[All Fields] OR "covid 19 nucleic acid testing"[MeSH Terms] OR "covid 19 serological testing"[All Fields] OR "covid 19 serological testing"[MeSH Terms] OR "covid 19 testing"[All Fields] OR "covid 19 testing"[MeSH Terms] OR "sars cov 2"[All Fields] OR "sars cov 2"[MeSH Terms] OR "severe acute respiratory syndrome coronavirus 2"[All Fields] OR "ncov"[All Fields] OR "2019 ncov"[All Fields] OR (("coronavirus"[MeSH Terms] OR "coronavirus"[All Fields] OR "cov"[All Fields]) AND 2019/11/01:3000/12/31[Date - Publication]))

Scopus

TITLE-ABS-KEY (covid-19) AND TITLE-ABS-KEY (("vitamin d" OR "ergocalciferols")) AND (LIMIT-TO (LANGUAGE , "English")) AND (LIMIT-TO (SRCTYPE , "j")) AND (EXCLUDE (DOCTYPE , "re") OR EXCLUDE (DOCTYPE , "le") OR EXCLUDE (DOCTYPE , "no") OR EXCLUDE (DOCTYPE , "ed") OR EXCLUDE (DOCTYPE , "er") OR EXCLUDE (DOCTYPE , "cp"))
